# Increased Incidence of Mitochondrial Cytochrome C Oxidase 1 Gene Mutations in Patients with Primary Ovarian Insufficiency

**DOI:** 10.1371/journal.pone.0132610

**Published:** 2015-07-30

**Authors:** Xiumei Zhen, Bailin Wu, Jian Wang, Cuiling Lu, Huafang Gao, Jie Qiao

**Affiliations:** 1 Center for Reproductive Medicine, Department of Obstetrics and Gynecology, Peking University Third Hospital, Beijing, 100191, China; 2 Genetic Diagnosis Lab Medicine, Boston Children’s Hospital, Harvard Medical School, Boston, Massachusetts, 02115, United States of America; 3 Human Genetic Resource Center, National Research Institute for Health and Family Planning, Beijing, 100081, China; 4 Shanghai Children's Medical Center, Shanghai Jiao Tong University School of Medicine, Shanghai, 200127, China; Institute of Zoology, Chinese Academy of Sciences, CHINA

## Abstract

Primary ovarian insufficiency (POI), also known as premature ovarian failure (POF), is defined as more than six months of cessation of menses before the age of 40 years, with two serum follicle stimulating hormone (FSH) levels (at least 1 month apart) falling in the menopause range. The cause of POI remains undetermined in the majority of cases, although some studies have reported increased levels of reactive oxygen species (ROS) in idiopathic POF. The role of mitochondrial DNA in the pathogenesis of POI has not been studied extensively. This aim of this study was to uncover underlying mitochondrial genetic defects in patients with POI. The entire region of the mitochondrial genome was amplified in subjects with idiopathic POI (n=63) and age-matched healthy female controls (n=63) using nine pair sets of primers, followed by screening of the mitochondrial genome using an Illumina MiSeq. We identified a total of 96 non-synonymous mitochondrial variations in POI patients and 93 non-synonymous variations in control subjects. Of these, 21 (9 in POI and 12 in control) non-synonymous variations had not been reported previously. Eight mitochondrial cytochrome coxidase 1 (*MT-CO1*) missense variants were identified in POI patients, whereas only four missense mutations were observed in controls. A high incidence of *MT-CO1* missense variants were identified in POI patients compared with controls, and the difference between the groups was statistically significant (13/63 vs. 5/63, *p*=0.042). Our results show that patients with primary ovarian insufficiency exhibit an increased incidence of mitochondrial cytochrome c oxidase 1 gene mutations, suggesting that *MT-CO1* gene mutation may be causal in POI.

## Background

Primary ovarian insufficiency (POI), also known as premature ovarian failure (POF), is defined as more than six months of cessation of menses before the age of 40 years, with two serum follicle stimulating hormone (FSH) levels (measured at least 1 month apart) in the menopause range. This multifactorial disease represents a public health concern, since it affects approximately1% of women under the age of 40. The incidence of POI in patients with primary amenorrhea is 10–28% and it is 4–18% in patients with secondary amenorrhea. Primordial oocytes are formed during fetal development and may reside within the ovaries for as long as 50 years before growth and development into mature oocytes. POI may develop as the result of a reduction in the number of congenital eggs or acceleration of the process of follicular atresia. Although POI is a heterogeneous disorder with a multifactorial etiology, including genetic, enzymatic, iatrogenic, immunological, and infectious disorders [1], the underlying cause of POI remains undetermined in the majority of cases.

Mitochondria are the energy-transducing organelles of eukaryotic cells, in which the fuels that drive cellular metabolism are converted into ATP through oxidative phosphorylation. Mitochondrial dysfunction has been associated with a wide range of human pathologies, including atherosclerosis, age-related neurodegenerative disease and human aging and infertility [2–6]. Mitochondrial energy production plays an important role in oogenesis and follicle maturation. Human oocytes contain the largest number of mitochondria, and oocytes of women with ovarian insufficiency have been reported to contain a lower mitochondrial DNA (mtDNA) copy number than women with a normal ovarian cells [7, 8]. Aging and age-related pathologies are frequently associated with a loss of mitochondrial function, mainly due to the accumulation of mtDNA mutations and deletions [6, 9]. Various studies have suggested oxidative stress as an etiopathological factor in reproductive disorders and increased levels of reactive oxygen species (ROS) are believed to be causative in idiopathic POF [4, 10, 11]. High ROS levels induce alterations in mitochondrial DNA, leading to mitochondrial dysfunction, which may play a role in POI by increasing the production of ROS [12]. Although mitochondrial energy production plays an important role in oogenesis, follicle maturation, ovulation and embryogenesis [9, 13, 14], the role of mitochondrial DNA in the pathogenesis of POI has not been studied extensively. The mitochondrial genome is contained in double-stranded, circular DNA, which, unlike nuclear DNA, does not contain histones or introns, making mtDNA more vulnerable to mutations and deletions. Whole exome sequencing is a powerful tool for detecting novel pathogenic mutations in patients with suspected mitochondria disease. The aim of this study was to uncover underlying mitochondrial genetic defects by sequencing the mitochondrial genome of patients with POI.

## Materials and Methods

### Subjects

Our study was comprised of 63 POI patients and 63 age-matched healthy females recruited from Peking University Third Hospital. Inclusion criteria for participation were: (1) age < 40 years, (2) amenorrhea for > 6 months, (3) FSH > 40IU/L, (4) chromosome karyotype 46 XX. Criteria for control subjects were: (1) age-matched with the POI group, (2) normal menstrual cycles, (3) normal hormone levels. Women with histories of chemotherapy, pelvic surgery, radiation exposure or smoking were excluded from the study. Pelvic ultrasound for assessment of ovarian size or follicular activity is routinely performed for determination of POI in our clinic. This study was approved by the institutional review board (IRB) of the Medical Department of Peking University, and all participants gave their written informed consent.

### DNA isolation and hormone level assessment

Peripheral blood samples were collected and DNA was extracted from 5 mL anti-coagulated whole blood using a Puregene DNA extraction Kit (Qiagen, Valencia, CA). Estradiol (E_2_), FSH, luteinizing hormone (LH), testosterone (T) and androgen (A) were measured in plasma specimens with Immulite (Siemens, Germany) according to the instructions of the manufacturer.

### Quantification of ATP

ATP content was measured using a commercial ATP assay kit and a luminometer (Bioluminat Junior; Berthold). The assay was based on theluciferin-luciferase reaction, and the manufacturer’s instructions were followed. This experiment was repeated at least three independent times.

### PCR amplification

The entire region of the mitochondrial genome was amplified in all POI patients and controls using nine primer pair sets [15]. Primers were synthesized by Integrated DNA Technologies (Coralville, IA).

Library preparation and massively parallel sequencing

Library preparation was performed according to the SureSelect^XT^ Target enrichment system for Illumina paired-end sequencing library (http://www.genomics.agilent.com/files/Manual/G3360-90020_SureSelect_IlluminaMultiplexed_1.1.1.pdf). Mitochondria DNA was sheared using a CovarisUltrasonicator (Covaris, MA). Adaptor-ligated libraries were constructed using Paired-End genomic DNA kits (Illumina,CA). The multiplexed samples were sequenced on the Illumina Miseq.

### Variant analysis and prioritization

Sequencing data were aligned to the human mitochondrial genome (GenBank: NC 012920) by NextGENe software (SoftGenetics, State College, PA). Variants were annotated based on human genome database included in Mitomap (http://www.mitomap.org) and MtDB (http://www.genpat.uu.se/mtDB/) were filtered out. Condel (http://bg.upf.edu/condel/home) was used to predict the pathogenicity of non-synonymous variants. Condel is a method to assess the outcome of non-synonymous single-nucleotide variants (SNVs) using a consensus deleteriousness score that combines the results of analysis by various tools (e.g. SIFT, PolyPhen-2, Mutation Assessor). Novel changes were further used PolyPhen-2 (Polymorphism Phenotyping v2;http://genetics.bwh.harvard.edu/pph2) which identifies changes that induce probable DNA damage. Relevant data are available in the European Nucleotide Archive, and the accession number is PRJEB9519.

### Statistical analysis

Quantitive data are expressed as mean ± SD. T-test was used to compare hormone serum levels, BMI and ovarian volume. All tests applied were two-tailed. The Pearson chi-squared test was used to determine significance in mtDNA gene mutations between POI patients and controls. The significance level was defined as *p*-value < 0.05. Statistical analysis was performed with the SPSS 11.5 package.

## Results

The characteristics of the POI patients and healthy subjects are outlined in Table 1. There were significant differences in FSH, LH, E2, T levels, ovarian volume and ATP levels between the two groups.

**Table 1:** Characteristics of women with POI and age-matched healthy women.

Parameter	POI (range) N = 63	Health (range) N = 63
Age (year)	27.6 ±4.51 (21–38)	27.5 ±4.57(21–39)
Menarche (age)	13.5 ±1.71 (12–18)	13.4±1.15 (12–15)
Amenorrhea (year)	6.33±4.71 (2–15)	0
BMI (kg/m2)	20.5±2.12 (16–27)	20.33±1.56 (18–25)
FSH (IU/L)	80.25±33.6 (44.7–140.2)*	7.6±1.74(5.35–9.7)
LH (IU/L)	38.65±14.8 (20.4–89.7)*	4.29±1.89(2.89–9.56)
E2 (pmol/L)	80.4±13.3 (73.4–109)*	136±53.9(96.9–240)
T (nmol/L)	0.781±0.29(0.69-)**	1.32±0.379()
A (nmol/L)	5.46±1.9(2.5–6.7)	6.02±1.97(4.3–8.9)
Ovarian volume#	1.512±2.01(0.489–7.53)*	13.1±1.61(9.63–19.8)
ATP level	1132.54±117.17(927.5–1299.5)***	1580.77±149.72(1354–1852.5)

* *p* = 0.000,

** *p* = 0.002,

***p = 0.0098

^#^ovarian volume was the average of volume of each ovaries. Ovarian volume was calculated using the formula for a prolate ellipsoid: longitudinal diameter × anterioposterior diameter × transverse diameter × 0.5233.

**Fig 1:**
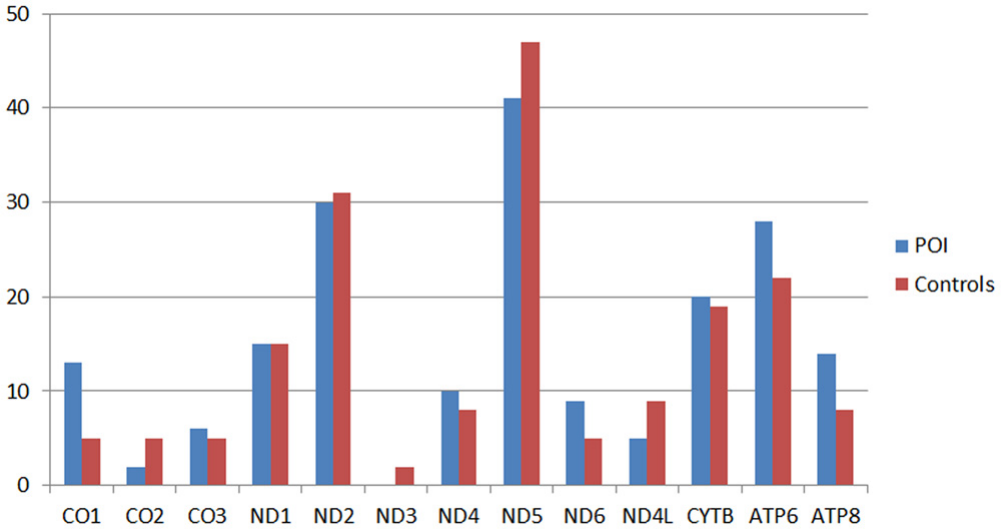
Nonsynonymous variations in different mitochondrial genes. The X axis was the mitochondrial gene name. The Y axis was the sample numbers which have nonsynonymous variations.

Synonymous variations have been routinely classified as innocuous polymorphisms and are assumed to be functionally neutral. Thus, we analyzed the non-synonymous variations observed in each group. A total of 96 non-synonymous variations were observed in POI patients and 93 non-synonymous variations in control subjects. Of these, 21 (9 in POI and 12 in control) non-synonymous variations had not been reported previously. The non-synonymous variations in different mitochondrial genes observed in the POI and control groups are shown in Fig 1.

The entire coding region of the gene for cytochrome coxidase subunit 1 (*MT-CO1* or *COX1*) was sequenced from each of the 63 POI patients and 63 controls. A total of 11 *MT-CO1* missense variants were identified, including five that had not been discovered previously (Tables 2 and 3). A high incidence of *MT-CO1* missense variants was observed in POI patients compared with controls, and the difference between the two groups was statistically significant (13/63 vs. 5/63, *p* = 0.042). The nucleotide changes *mt-CO1* c.790A>G and c.802T>C, which have not been reported previously, were predicted to be deleterious by Condel, a tool used to predict the effect of amino acid substitutions. The changes were analyzed again using PolyPhen-2, another tool used to predict possible impact of amino acid substitutions, and again identified as alterations causing probable DNA damage. These position in the mitochondria genome are highly conserved among all respiring organisms. At position 7068, the nucleotide change *MT-CO1* c.1165A>G was identified in two POI patients, but not in controls. This change was predicted to be possibly damaging with a score of 0.868 by PolyPhen-2, whereas it was predicted to have a neutral effect by Condel. Another change,*MT-CO1* c.667G>T, which had not been previously reported, was predicted by Condel to be neutral.

**Table 2:** *MT-CO1* missense mutations in POI patients.

No.	Position	Nucleotide change	Homogeneity/Heterogeneity	Amino acid change	PolyPhen-2	Condel	Previously reported	Frequency
1	6693	c.790A>G	Hetero	264K>KE	Probably damaging/1.000	Deleterious/0.699	No	1/63
*2*	6705	c.802T>C	Hetero	268F>LF	Probably damaging/0.999	Deleterious/0.761	No	1/63
3	6570	c.667G>T	Homo	223A>S	Benign/0.009	Neutral/0	No	1/63
4	7068	c.1165A>G	Homo	389I>V	Possibly damaging/0.868	Neutral/0.038	No	2/63
5	7270	c.1367T>C	Hetero	456V>AV	Benign/0.054	Neutral/0.002	Yes[24]	3/63
6	6253	c.350T>C	Homo	117M>T	Benign/0.000	Neutral/0	Yes[24]	3/63
7	7389	c.1486T>C	Homo	496Y>H	Benign/0.000	Neutral/0	Yes[24]	1/63
8	7129	c.1226A>G	Homo	409Y>C	Benign/0.003	Neutral/0.001	Yes[25]	1/63

**Table 3:** *MT-CO1* missense mutations in control subjects.

No	Position	Nucleotide change	Homogeneity/Heterogeneity	Amino acid change	PolyPhen-2	Condel	Previously reported	Frequency
1	6662	c.759A>T	Homo	253M>I	Benign/0.003	Neutral/0	No	1/63
2	6253	c.350T>C	Homo	117M>T	Benign/0.000	Neutral/0	Yes[24]	2/63
3	6285	c.382G>A	Homo	128V>I	Benign/0.066	Neutral/0.032	Yes[26]	1/63
4	6366	c.463G>A	Homo	155V>I	Benign/0.000	Neutral/0	Yes[27]	1/63

Aside from the *MT-CO1* gene, there were no significant differences in other genes between the two groups. Table 4 presents the novel mt-DNA sequence variants detected in POI patients and control subjects.

**Table 4:** Novel non-synonymous variation in other mitochondrial DNA genes.

No.	Position	Gene	Nucleotide change	Homogeneity/heterogeneity	Amino acid change	Condel	Patients(P)/Control(C)	Frequency
1	9423	COX3	c.217C>T	Homo	73P>S	Neutral/0.015	P	1/63
2	12865	ND5	c.529A>G	Homo	177I>V	Neutral/0	P	1/63
3	12939	ND5	c.603A>C	Homo	201M>I	Neutral/0	P	1/63
4	14576	CYTB	c.10A>G	Homo	4M>V	Neutral/0.004	P	1/63
5	15398	CYTB	c.652A>G	Homo	218I>V	Neutral/0	P	1/63
6	7749	COX2	c.164T>C	Homo	55I>T	Neutral/0.001	C	1/63
7	3548	ND1	c.242T>C	Homo	81I>T	Neutral/0.008	C	1/63
8	3355	ND1	c.49A>G	Homo	17M>V	Neutral/0.015	C	1/63
9	4794	ND2	c.325G>A	Hetero	109A>TA	Neutral/0.015	C	1/63
10	12068	ND4	c.1309A>G	Hetero	437M>MV	Neutral/0.01	C	1/63
			c.1309A>G	Homo	437M>V		C	1/63
11	14116	ND5	c.1780C>T	Homo	594P>S	Neutral/0.005	C	1/63
12	13858	ND5	c.1522A>G	Homo	508T>A	Neutral/0	C	1/63
13	15222	CYTB	c.476A>G	Homo	159D>G	Neutral/0.064	C	1/63
14	14880	CYTB	c.134T>C	Homo	45I>T	Neutral/0.018	C	1/63
15	8897	ATP6	c.371C>T	Homo	124A>V	Neutral/0	C	1/63
16	8945	ATP6	c.419T>C	Homo	140M>T	Neutral/0.024	C	1/63

## Discussion

In this study, a total of 96 non-synonymous variations in mitochondrial DNA were observed in POI patients and 93 non-synonymous variations in control subjects. There were eight non-synonymous variations of the *MT-CO1* gene in 13 POI patients whereas only four non-synonymous variations of the *MT-CO1* gene were observed in five control subjects.

Primary ovarian insufficiency occurs due to an inadequate initial pool of primordial follicles and accelerated follicle apoptosis. Adequate ATP is necessary for germ cell growth, development and apoptosis during oogenesis. An investigation showed that defects in mitochondrial biogenesis or/and insufficient mitochondrial mass are associated with the failure in oocyte maturation and abnormal embryo development. The mitochondrial genome must be replicated with great accuracy because mitochondria are inherited by the zygote exclusively from the oocyte. Decrease in mitochondrial activity has been reported to impair the oocyte maturation. Numerous studies have reported that oxidative stress is an underlying mechanism in female reproductive disorders [10, 11], and that nutrient supplementation (CoQ10) can improve the quality of oocyte and embryos in older patients [16]. Dysfunction of cytochrome coxidase affects cellular energy metabolism and causes increased production of reactive oxygen species with a variety of deleterious consequences in humans [5]. Mutation in the human *MT-CO1* gene has been associated with several diseases, including neurodegenerative disease. To our knowledge, no reports focusing on the relationship between *MT-CO1* mutations and POI have been published. In this study, we found that the frequency of *MT-CO1* missense mutations in POI patients was higher than in control subjects (13/63 vs. 5/63, *p*<0.042).

Cytochrome c coxidase (COX) or complex IV is the third and final enzyme of the electron transport chain of mitochondrial oxidative phosphorylation. It collects electrons from reduced cytochrome c and transfers them to oxygen to produce water. Also, it has been demonstrated that *MT-CO1* is the terminal component and one of the three genomic components of the mitochondrial respiratory chain. Another study has shown that *MT-CO1* can be considered as an indirect indicator of activity and quantity of mtDNA. It is the major site of cell oxygen consumption and plays a fundamental role in energy production in aerobic cells (OMIM, http://omim.org/entry/516030). Two nucleotide changes, *MT-CO1* c.790A>G and *MT-CO1*c.802T>C, which have not been reported previously, were identified and predicted to be deleterious to the *MT-CO1* gene by Condel and PolyPhen-2. These point mutations result in the substitution of aglutamic acid (E) in place of lysine (K) at position 264, and a leucine (L) in place of phenylalanine (F) at position 268. These positions of mitochondria genomic are highly conserved among all respiring organisms. In position 7068, the mutation *MT-CO1* c.1165A>G, which has not been reported previously, was identified in two POI patients but not in control subjects, and was predicted to be damaging by PolyPhen-2 but neutral by Condel. This point mutation leads to a valine (V) in place ofisoleucine (I) at position 389. Another homoplasmic variant, *MT-CO1* c.667G>T, which has not been previous reported, leads to the substitution of a serine (S) in place of alanine (A) at position 223. This study revealed a significantly higher incidence of non-synonymous variations of *MT-CO1* in patients with POI compared to healthy control subjects. The over expression levels of *MT-CO1* gene are related to the oocyte maturation [6, 17]. *MT-CO1* gene mutations reduce COX activity and produce low ATP. Decrease in ATP synthesis is correlated with accumulation of calcium ions in cells, dysfunction of mitochondria, and increasing apoptotic activity [18]. As we know, Intra-oocyte PI3K/mTOR pathways have been indicated to play a central role on the activation of primordial follicles [19, 20]. Low ATP could activate mTOR and the pool of primordial follicles is activated prematurely due to elevated mTORC1 activity in oocytes [21, 22], which will accelerate the ovarian follicle development and rate of follicle loss. This results in depletion of follicles in early adulthood, causing premature ovarian failure (POF). Therefore, may lead to impaired oogenesis and low primordial follicle production or accelerated follicle depletion [23].

In summary, we found a high incidence of *MT-CO1* missense mutations in patients with idiopathic primary ovarian insufficiency and identified two novel missense mutations in the mitochondrial cytochrome c oxidase 1 gene that were predicted to be damaging. Thus, *MT-CO1* gene mutations may be an important causal event in the development of POI.
